# Macrognathia Secondary to Renal Osteodystrophy in a Hemodialysis Patient: A Case Report and Literature Review

**DOI:** 10.3390/jcm15218256

**Published:** 2026-10-23

**Authors:** Mohamed Jaber, Mohamed S. Ingagou, Abdullah M. Elameen, Prathibha Prasad, Mehzabin Ahmed

**Affiliations:** 1 Clinical Dental Sciences Department, College of Dentistry, Ajman University, Ajman P.O. Box 346, United Arab Emirates; m.ingagou@ajman.ac.ae (M.S.I.); abodxr13@gmail.com (A.M.E.); 2 Center for Medical and Bio-Allied Health Sciences Research, Ajman University, Ajman P.O. Box 346, United Arab Emirates; p.prasad@ajman.ac.ae; 3 Basic Medical and Dental Sciences Department, College of Dentistry, Ajman University, Ajman P.O. Box 346, United Arab Emirates; 4 Biomedical Sciences Department, College of Medicine, Gulf Medical University, Ajman P.O. Box 4184, United Arab Emirates

**Keywords:** hyperparathyroidism, secondary, macrognathia, oral manifestations, renal dialysis, renal osteodystrophy, osteitis fibrosa cystica

## Abstract

**Background**: Renal osteodystrophy (ROD) is a complex metabolic bone disorder encompassing the full spectrum of skeletal abnormalities arising from chronic kidney disease–mineral and bone disorder (CKD-MBD). Although ROD is a well-recognized systemic complication of end-stage renal disease (ESRD), its manifestation as progressive maxillofacial enlargement macrognathia is rare and may precede recognition of the underlying nephropathy in clinical dental settings. The paper aims to report a case of ROD-associated macrognathia in a patient on long-term hemodialysis and a contemporary literature review. **Case Presentation**: We describe a 45-year-old female patient on long-term maintenance hemodialysis for ESRD secondary to Grade I bilateral nephropathy who presented to our dental clinic with a 6-month history of progressive, painless diffuse swelling of the mandibular anterior region that subsequently involved the hard palate. Oral examination revealed bilateral jaw enlargement, severe generalised periodontitis, xerostomia, uremic fetor, and hyposalivation. Panoramic radiography demonstrated ground-glass trabecular changes, cortical thinning, and loss of the inferior cortex. Histopathology of an incisional biopsy revealed features of fibrous replacement of medullary bone, irregular osteoid mineralization, increased osteoblastic activity, and osteoclastic resorption lacunae suggestive of osteitis fibrosa cystica. **Conclusions**: This case illustrates the importance of the dental clinician’s role in recognizing the oral and maxillofacial manifestations of systemic metabolic disease. Macrognathia should be included in the differential diagnosis of jaw enlargement in any patient with a history of CKD or ESRD. Multidisciplinary collaboration between dentists, oral pathologists, and nephrologists is essential for timely diagnosis and optimal management.

## 1. Introduction

Chronic kidney disease (CKD) is diagnosed when structural and functional abnormality lasting longer than 3 months has detrimental health consequences, especially cardiovascular complications [1]. The global burden of CKD has more than doubled over the last 3 decades to reach 788 million in 2023 [2]. Long-term renal replacement therapy, most commonly as maintenance hemodialysis, is required when end-stage renal disease (ESRD) progresses. This therapy itself causes a complex range of systemic sequelae, caused by the release of factors by the disease and repair processes in the kidney. One such factor, Activin A, is responsible for increased osteoclastic proliferation and activity resulting in increased bone turnover. This results in the clinical syndrome of CKD-mineral and bone disorder (CKD-MBD) [3]. The bone consequences of CKD, termed as renal osteodystrophy (ROD), are currently being expanded to include vascular complication [4]. ROD is an umbrella term that includes all the skeletal abnormalities resulting from CKD, such as osteitis fibrosa cystica (increased osteoclastic activity due to secondary hyperparathyroidism), osteomalacia (impaired mineralization), adynamic bone disease (suppressed bone formation due to downregulation of parathyroid hormone [PTH]), osteoporosis, and increased bone fragility and mixed uremic osteodystrophy [4,5]. Bone biopsies serve as an accurate diagnostic tool and are critical as a guide for the optimal management of CKD-MBD [6]. Additionally, vascular and soft-tissue calcifications, left ventricular hypertrophy, endocrine and immune disorders, neurobehavioral manifestations, and erythropoietic abnormalities may also be seen as complications of CKD-MBD [7].

Derangements in calcium, phosphorus, parathormone vitamin D metabolism, and fibroblast growth factor-23 (FGF-23) underscore the abnormalities in bone turnover and mineralization that manifest as the various forms of ROD and skeletal fractures [4,7,8]. Reduced renal phosphate excretion in CKD causes fibroblast growth factor 23 (FGF23) to gradually increase and Klotho, its co-receptor, to be suppressed. This impairs calcitriol synthesis, decreases intestinal calcium absorption, and eventually stimulates chronic PTH hypersecretion from the parathyroid glands [7,9,10]. This pathophysiologic cascade of CKD-MBD and the resultant hyperparathyroidism determines the acceleration in the rate of bone resorption, and fibrosis in the hematopoietic marrow [7]. An uncommon oral and maxillofacial manifestation of CKD-MBD are the brown tumors of hyperparathyroidism in the jaw, and these may be associated with life-threatening complications [11].

Delayed tooth eruption, hypoplastic enamel abnormalities, abnormal lamina dura, periodontal destruction, xerostomia, uremic halitosis, and mucosal pallor are among the oral and maxillofacial involvement in ROD that has been extensively documented in the dental literature [12,13]. However, it is uncommon for considerable progressive jaw growth macrognathia to be the presenting symptom in a dental environment [14,15,16]. A significantly severe facial manifestation of CKD, known as Sagliker syndrome (SS), results from uncontrolled secondary hyperparathyroidism. SS patients develop maxillary and mandibular overgrowth, destruction of nasal bones and cartilages, wide spacing of teeth, and soft tissue tumors in the oral cavity, along with hearing loss and neurological and psychological features [17].

Brown tumors occur in 1.5% of CKD with a majority of these occur in dialysis-dependent patients with untreated secondary hyperparathyroidism [18]. However, significant progressive jaw enlargement macrognathia as the presenting complaint in a dental setting is rare [14,15]. Published literature in English describing ROD-associated macrognathia, is scant since the seminal multicenter series involving dialysis-dependent patients with uncontrolled secondary hyperparathyroidism [16].

The authors report a case of ROD-associated macrognathia in a 45-year-old woman on long-term hemodialysis, diagnosed by incisional biopsy, and discuss the clinical, radiographic, and histopathological features alongside a contemporary literature review. This case underscores the critical role of oral healthcare providers in recognizing maxillofacial presentations of systemic metabolic disease and reinforces the need for integrated multidisciplinary management.

## 2. Case Presentation

### 2.1. Medical and Dental History

A 45-year-old female patient presented to the Department of Oral Medicine complaining of a 6-month history of progressive, painless swelling of the anterior mandibular region, and, more recently, similar involvement of the hard palate. She reported difficulty with mastication but denied spontaneous pain, paresthesia, or constitutional symptoms.

The patient had a 15-year history of chronic kidney disease secondary to bilateral grade I reflux nephropathy. She progressed to end-stage renal disease in 2019; at presentation, she had been receiving maintenance hemodialysis three times weekly for 5 years. Each session lasted 4 h; the most recent single-pool Kt/V was 1.45 (urea reduction ratio 72%). Essential hypertension was diagnosed approximately 1 year before presentation. She was prescribed a regimen of a loop diuretic, an amlodipine-based calcium channel blocker, calcitriol (0.25 µg daily), and a multivitamin supplement. Her maintenance medication included an erythropoiesis-stimulating agent (epoetin alfa 4000 IU intravenously three times weekly after dialysis), intravenous iron (iron sucrose 100 mg weekly), oral sodium bicarbonate (650 mg three times daily) for metabolic acidosis, a phosphate binder (sevelamer carbonate 800 mg with meals), 0.25 µg of calcitriol daily, a loop diuretic (40 mg of furosemide twice daily), amlodipine (5 mg daily), and a renal multivitamin supplement. Her dietary plan followed renal dietary recommendations for maintenance hemodialysis. Dietary potassium and phosphate restriction, sodium and fluid restriction to limit interdialytic weight gain, and an adequate protein intake of 1.0 to 1.2 g/kg/day was advised [19]. Family and personal history were not significant.

### 2.2. Clinical Examination

General examination revealed generalised pallor of the skin, mucous membranes, and conjunctivae, consistent with the anemia of chronic kidney disease. Extraorally, bilateral labial and buccal expansion of the alveolar ridges produced clinically evident enlargement of both the maxilla and mandible (macrognathia). The swelling was diffuse, firm, non-compressible, and non-tender on palpation, with no regional lymphadenopathy or overlying skin changes.

Intraorally, oral hygiene was poor with heavy generalised supragingival and subgingival calculus deposits. Uremic fetor was present, and the tongue blade test was positive for xerostomia, confirming hyposalivation. Mucosal examination revealed generalised erythema of the labial and buccal mucosa and diffuse generalised gingival inflammation. Periodontal assessment demonstrated widespread recession, pseudopockets, and pathological periodontal pocket formation. The alveolar mucosa overlying the swelling was intact, with normal surface texture and coloration, and no evidence of ulceration, sinus, or fluctuation.

**Figure 1:**
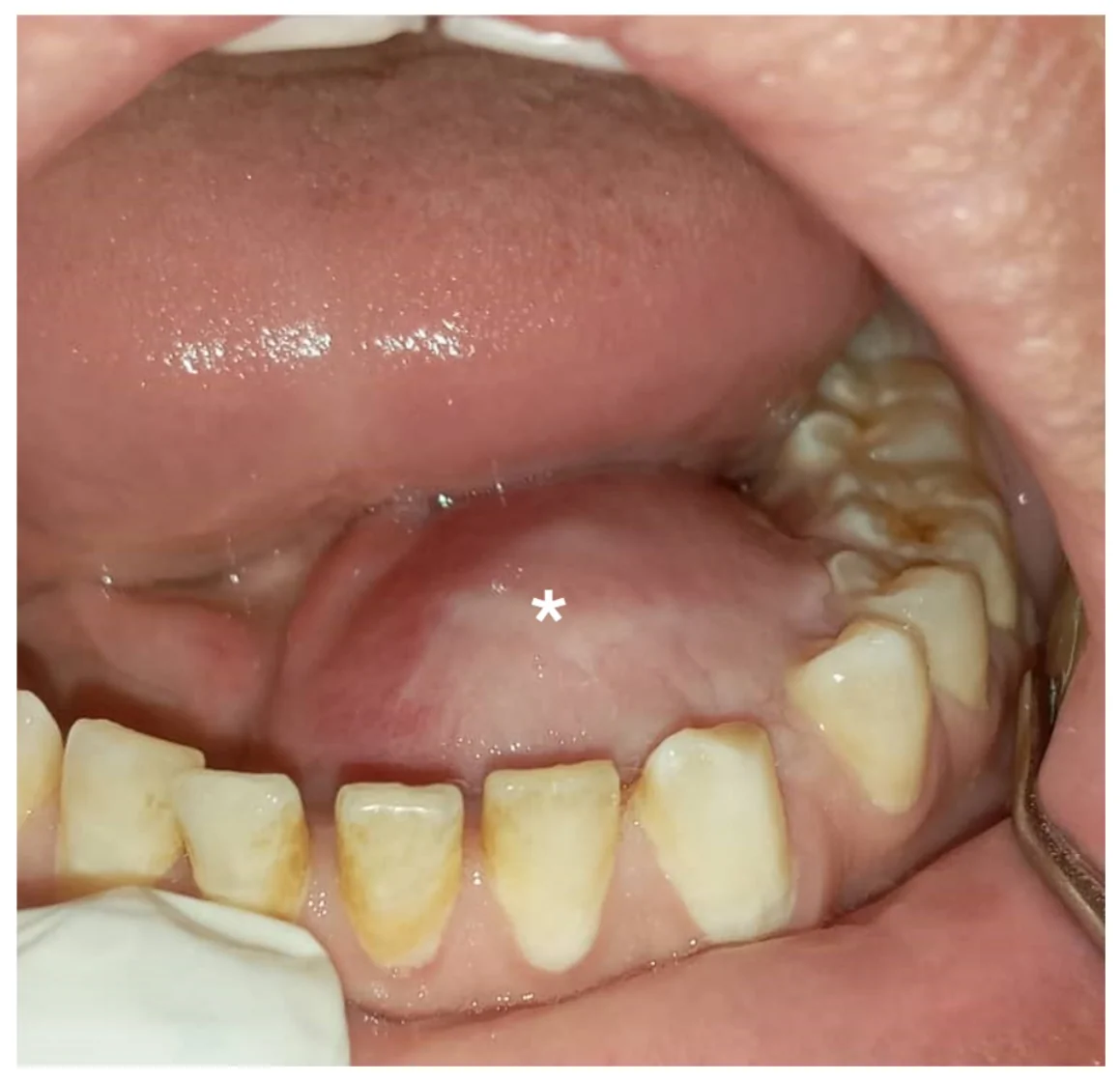
Non-tender mandibular swelling appears as a diffuse, firm, and non-compressible on palpation (asterisk).

**Figure 2:**
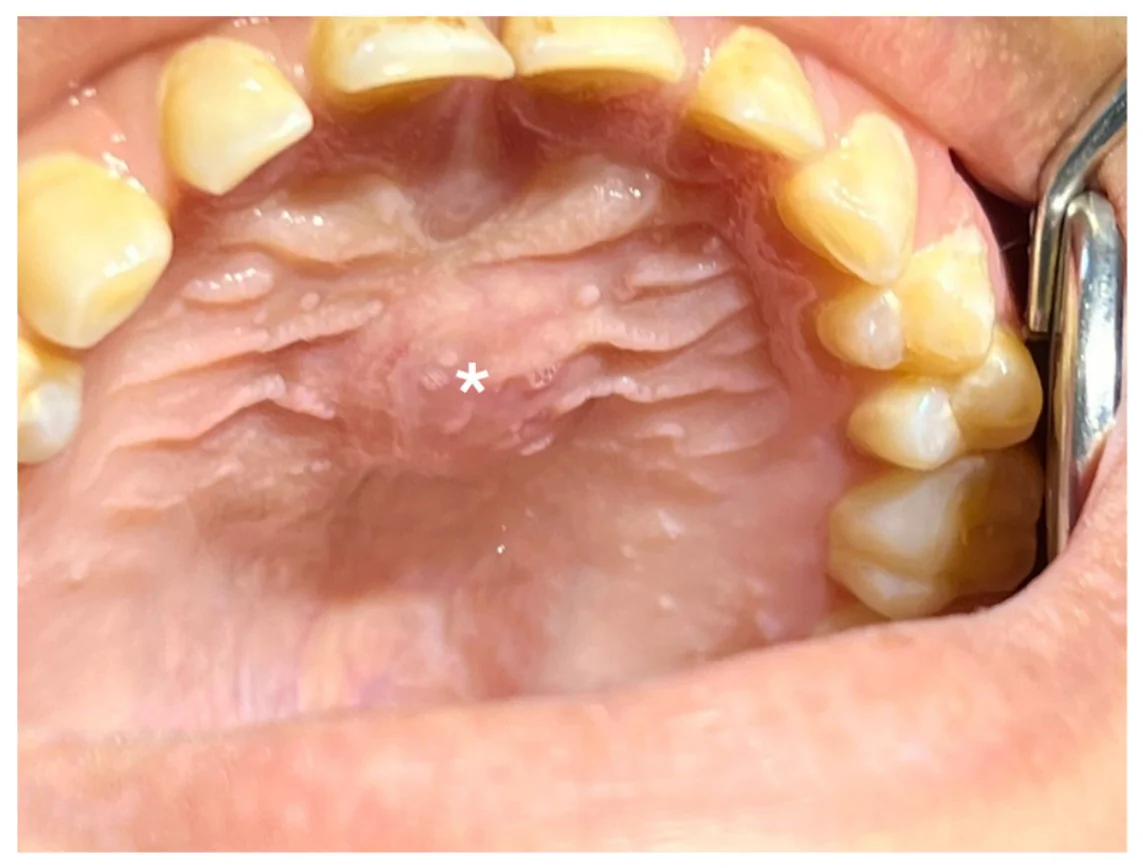
Maxillary lesion seen as a sessile nodular and firm midline swelling on the hard palate (asterisk).

A discrete, firm, sessile nodular expansion was also present on the hard palate in the midline, consistent with palatal involvement by the same disease process. (Figure 1 and Figure 2).

### 2.3. Radiographic Findings

**Figure 3:**
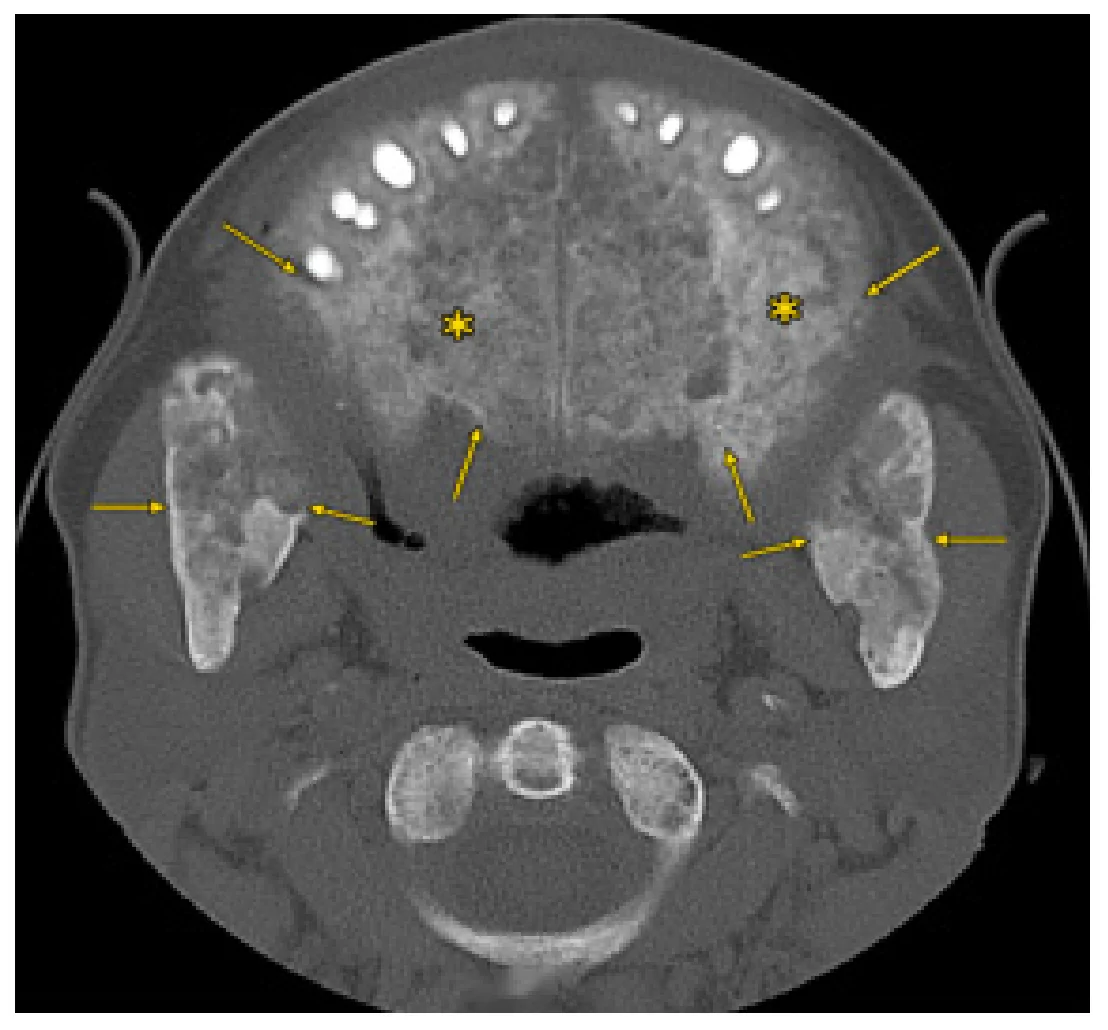
Cone beam computed tomography (CBCT) findings: Axial section showing buccal and lingual cortical expansion (arrows), cortical thinning, and a homogeneous ground-glass trabecular pattern (asterisk).

**Figure 4:**
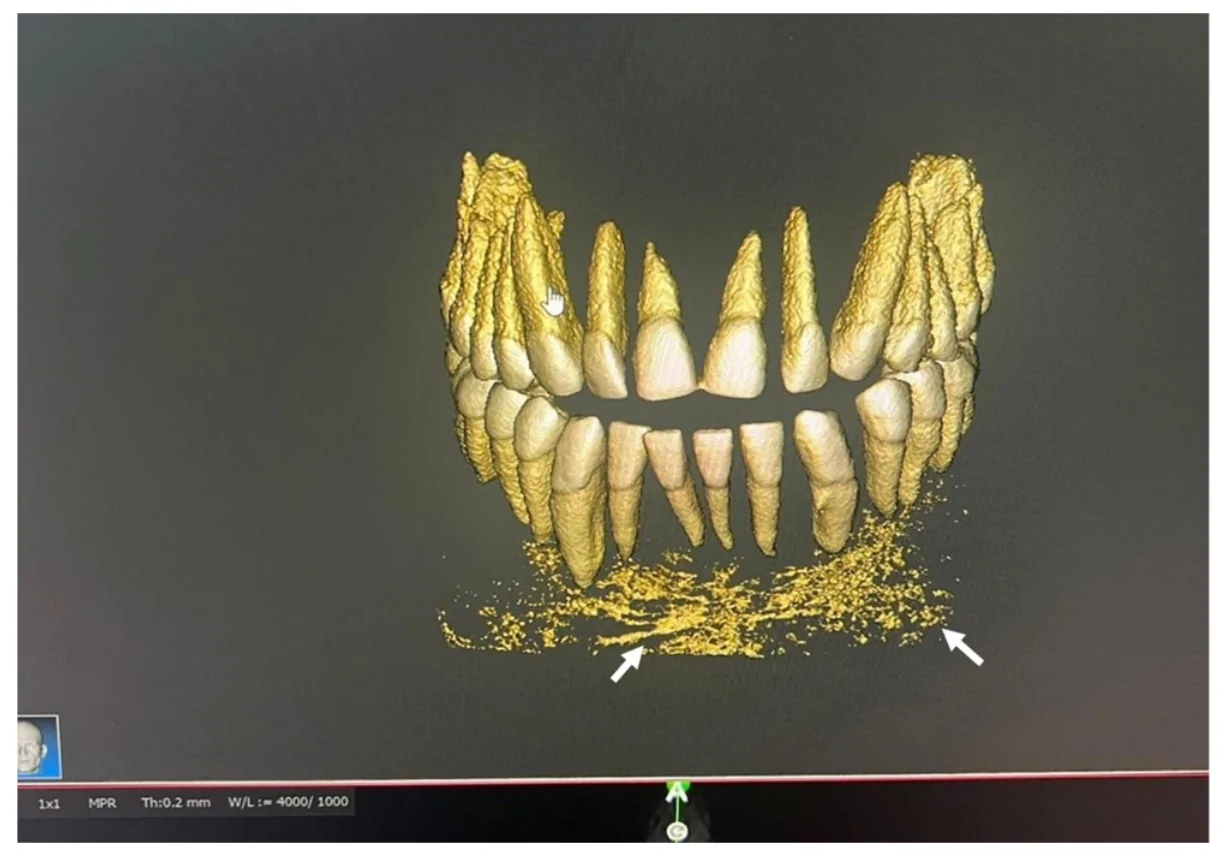
3D- CT reconstruction showing upper and lower jaws with teeth and expansile lesion in the mandibular bone (indicated by the white arrows).

CBCT and digital panoramic radiograph (orthopantomogram) demonstrated widespread changes throughout the mandible and maxilla, with a generalized reduction in radiodensity in both affected bones. Diffuse, bilateral expansion of the maxillary alveolar process and palate crosses the midline. The internal architecture is replaced by a fine, uniform, ground-glass density (asterisks in Figure 3) with loss of normal trabecular and cortical distinction. The buccal and palatal/lingual cortical plates are expanded and markedly thinned (arrows in Figure 3) but remain intact, with no perforation or periosteal reaction. Teeth roots and developing teeth are embedded within the lesion and displaced along the expanded arch; no resorption is evident at this level. Both mandibular rami are expanded buccolingually with thinned cortices (arrows in Figure 3); their internal pattern is more heterogeneous, mixing ground-glass and lucent areas. The posterior maxillary expansion encroaches on the nasopharyngeal airway; the overlying soft tissues appear normal. Cervical spine C1 and the dens are normal, serving as an internal reference for normal bone density. Generalised loss of the lamina dura was identified around all remaining teeth. The combination of cortical thinning, loss of lamina dura, and granular ground-glass trabeculae is radiographically suggestive of osteitis fibrosa cystica in the context of secondary hyperparathyroidism (Figure 3 and Figure 4).

### 2.4. Differential Diagnosis

The ground-glass trabecular pattern is not specific to ROD and overlaps radiographically with several other metabolic and dysplastic bone conditions. The differential diagnosis considered at initial assessment included: (1) Diffuse ROD-associated macrognathia; (2) Localized brown tumor; (3) Fibrous dysplasia; (4) Central giant cell granuloma; (5) Ossifying fibroma; (6) Paget’s disease; (7) Malignant bone neoplasm. (Table 1).

Sagliker syndrome is a severe craniofacial deformity phenotype described in longstanding, poorly controlled secondary hyperparathyroidism of chronic renal failure [1]. Our patient showed the craniofacial component of Sagliker syndrome but not the full syndrome, as fingertip changes, short stature, hearing loss, and neuropsychological features were absent. We therefore classified the presentation as ROD-associated macrognathia, which may represent an early or incomplete form on the same spectrum (Table 2) [20].

### 2.5. Laboratory Investigations

The laboratory values at three time points: at presentation, after 3 months of the modified nephrology regimen, and at the latest follow-up (12 months).

The intact PTH of 1200 pg/mL (12 times the upper reference limit), as shown in Table 3, confirmed severe secondary hyperparathyroidism, above the KDIGO target of 2–9 times the upper limit for dialysis patients. Correlation with serum biochemistry (calcium, phosphate, intact PTH, alkaline phosphatase, Vitamin D status) and renal function is essential to differentiate entities mentioned in Table 1, since the jaw imaging findings alone are rarely pathognomonic. The combination of bilateral symmetric jaw enlargement, hyposalivation, uremic fetor, ground-glass radiographic changes, and biochemical changes, and a history of long-term hemodialysis, made ROD the primary working diagnosis, which was referred for histopathological confirmation to exclude a neoplasm.

### 2.6. Histopathological Findings

**Figure 5:**
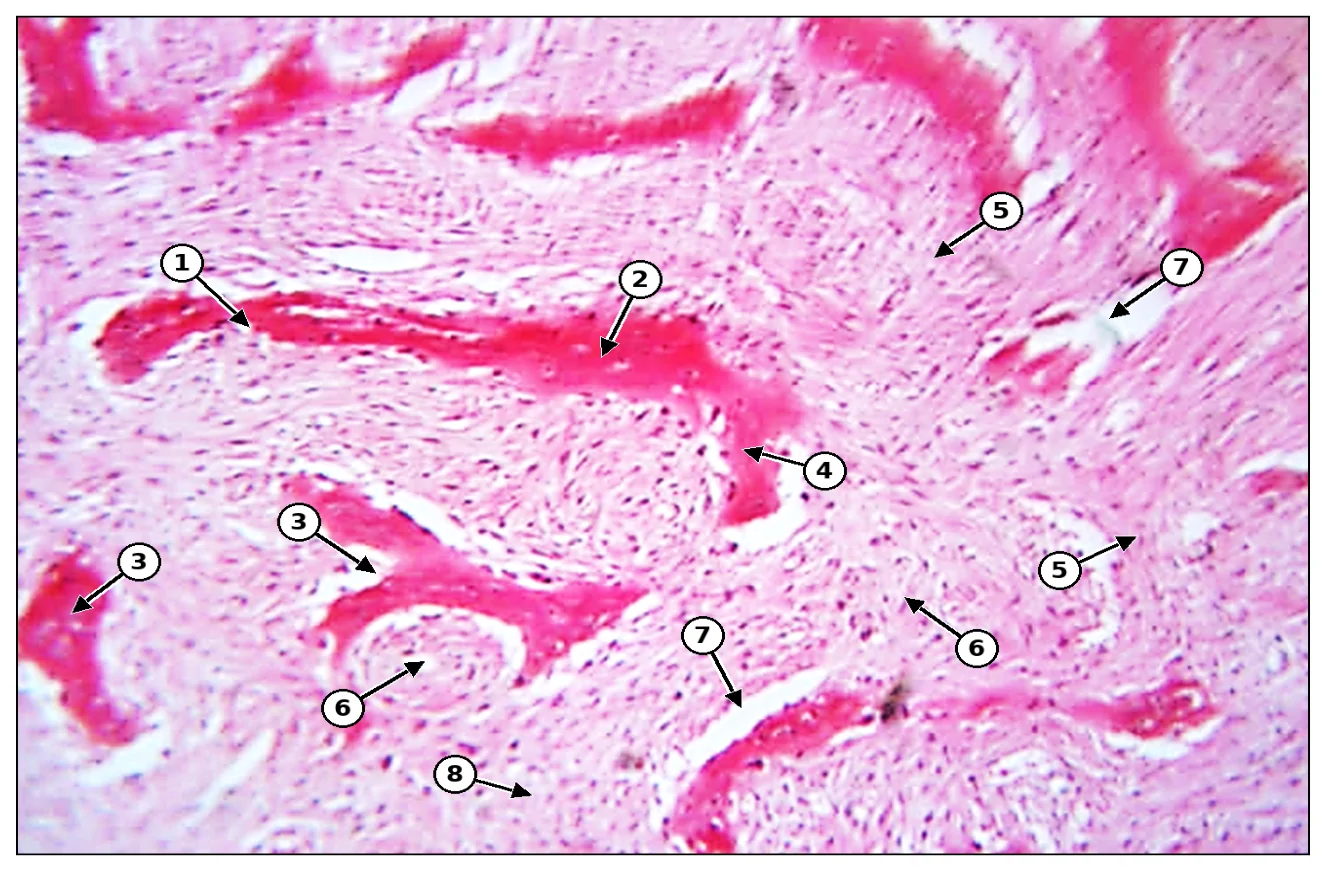
Histopathologic findings (H&E)-Bone trabeculae (1) of irregularly mineralized woven bone (2), seen as uneven patchy eosinophilia within the trabeculae. Some trabeculae appear as curved/anastomosing trabeculae C-shaped and interconnecting bony profiles (3). Trabeculae lack osteoblastic rimming and arise directly from the metaplastic stroma (4). Stroma consists of collagenous matrix with spindle fibroblasts (5) arranged in a storiform/whorled pattern (6) normal hematopoietic marrow, with interspersed. Retraction artefacts are seen as clefts separating bone from surrounding stroma (7). Hematopoietic marrow elements are replaced by fibrous tissue (8).

An incisional biopsy was performed on the day after dialysis under local anesthesia with 2% lidocaine with 1:100,000 epinephrine (Xylocaine), given as an inferior alveolar nerve block and labial infiltration. A mucoperiosteal flap was raised over the labial aspect of the anterior mandible with a No. 15 scalpel blade and a periosteal elevator. A bone specimen of approximately 10 × 8 × 5 mm was removed with a trephine bur under sterile saline irrigation. Haemostasias was achieved with oxidized cellulose, and the flap was closed with 4-0 polyglactin 910 sutures. The specimen was fixed in 10% neutral buffered formalin and decalcified in EDTA. On gross examination, the specimen was firm and greyish white. Prepared histopathologic sections were stained with hematoxylin and eosin. Microscopic examination revealed a cellular fibrous stroma replacing normal hematopoietic marrow, with interspersed trabeculae of irregularly mineralized woven bone. Osteoid seams were widened and showed irregular mineralization. There was marked osteoblastic rimming of bony trabeculae and prominent osteoclastic resorption with the formation of Howship’s lacunae. Multinucleated osteoclast-like giant cells were identified in several areas. Foci of marrow fibrosis with hemorrhage were also noted (Figure 5).

These features of fibrous replacement of marrow, woven bone trabeculae with irregular mineralization, osteoblastic rimming, and osteoclastic resorption lacunae are the hallmark histopathological findings of osteitis fibrosa cystica: the high-turnover subtype of renal osteodystrophy secondary to sustained PTH hypersecretion [7,21,22]. Histopathology, combined with clinical, radiographic, and systemic findings, was suggestive of renal osteodystrophy.

### 2.7. Management

After the diagnosis, a team of specialists in oral medicine, nephrology, and endocrinology met to discuss the case. To estimate the extent of mineral and hormonal disruption, biochemical analyses of serum intact PTH, calcium, phosphate, alkaline phosphatase, and 25-hydroxyvitamin D were performed. In compliance with the KDIGO 2017 CKD-MBD guidelines, the nephrology team evaluated and modified the patient’s calcimimetic/vitamin D analog regimen and dialysate calcium concentration [23]. Cinacalcet was initiated at 30 mg daily and titrated to 60 mg daily; 0.025 µg daily of oral calcitriol were switched to 1 µg daily of oral paricalcitol three times weekly; dialysate calcium was changed from 1.25 to 1.50 mmol/L; and sevelamer carbonate was increased from 800 mg to 1600 mg with meals.

Symptomatic relief, infection control, and palliative care were the main goals of oral management. A structured thorough oral hygiene program was planned, in coordination with the hemodialysis schedule. This included supragingival and deep subgingival debridement. The oral treatment was performed the day after dialysis to minimize the risk of bleeding from heparin anticoagulation. Chlorhexidine 0.2% mouthrinse was prescribed twice daily. Topical fluoride was applied to protect against caries risk exacerbated by xerostomia and hyposalivation. Salivary substitutes were recommended to manage xerostomia. Prophylactic oral antifungal therapy was discussed given the high risk of secondary candida superinfection in immunocompromised patients with xerostomia [24].

The patient was counselled that significant jaw enlargement secondary to ROD rarely fully regresses following medical management alone, and that surgical parathyroidectomy, if indicated by the nephrologist on the basis of refractory biochemical hyperparathyroidism, represents the only intervention associated with documented regression of jaw enlargement in case reports [25,26]. The patient was enrolled in a regular dental recall program with follow-up reviews scheduled at three-month intervals.

### 2.8. Follow Up and Outcome

Laboratory investigations were carried out in the three monthly follow-up visits. During 12 months of follow-up, intact PTH fell from 1200 to 210 pg/mL. A comparison of results at three time points, at presentation, after 3 months of the modified nephrology regimen, and at the latest follow-up (12 months), are included in Table 4.

The jaw enlargement had only slightly reduced on clinical examinations and on repeat panoramic radiograph. Periodontal inflammation and bleeding on probing had improved with the structured hygiene program. Parathyroidectomy was recommended but the patient declined, and medical management was continued.

## 3. Discussion

This case illustrates the rare but clinically important maxillofacial presentation of renal osteodystrophy in a patient receiving long-term maintenance hemodialysis. Although ROD affects the majority of patients with end-stage renal disease [3], presentation as progressive bilateral jaw enlargement is encountered infrequently, and its documentation in the dental literature remains limited to isolated case reports and small case series [25,26,27]. The case is clinically instructive because it brings together systemic disease, characteristic jaw changes, and histopathological findings, features that require coordinated interpretation rather than reliance on any single diagnostic test.

### 3.1. Pathophysiology of CKD-MBD and Jaw Involvement

The contemporary understanding of CKD-MBD pathophysiology centers on the FGF23–Klotho axis.

As functional renal mass declines, phosphate excretion is impaired, driving early and sustained elevation of FGF23, a phosphaturic hormone produced by osteoblasts and osteocytes [8,9]. FGF23 reduces calcitriol synthesis in healthy kidneys by promoting phosphaturia and suppressing renal 1α-hydroxylase activity through FGFR1/Klotho complexes [7,9,10]. This feedback system is attenuated in CKD by a progressive insufficiency of Klotho, which further leads to calcitriol deficiency, followed by an impaired calcium absorption from the intestine. Ensuing disturbance in calcium, phosphate, and vitamin D metabolism promotes secondary hyperparathyroidism. [9,11].

Osteitis fibrosa cystica or secondary hyperparathyroidism is a high-turnover bone disease caused by a chronic hypersecretion of PTH. In severe conditions, elevated PTH triggers RANK/RANKL signaling, which promotes rapid bone resorption, fibrous replacement of medullary space, and the development of reparative, non-neoplastic brown tumors [21,22]. Hemorrhages with accumulation of hemosiderin within the vascularized fibrous tissue contribute to its reddish-brown color [28]. Because of their rich hematopoietic red marrow, the flat bones of calvarium and facial skeleton seem to be particularly susceptible to these changes, which may account for the propensity of ROD to affect the jaw bones when the condition is unchecked [27,29]. The woven bone and fibrous tissue formed during this resorptive process can gradually build up, resulting in a solid, expansile jaw or macrognathia, which is evident in our patient and in the few numbers of cases that have been documented in the literature [25,26,27].

### 3.2. Oral and Maxillofacial Manifestations of CKD

Oral symptoms of chronic kidney disease (CKD) are varied and include both direct uremic effects and treatment-associated side effects [12,13]. A recent comprehensive systematic review by García-Rios et al. (2025) identified hyposalivation, increased calculus index, periodontal disease, enamel defects, and alterations in salivary composition as the most consistently reported oral findings in CKD patients [12]. Xerostomia in this population arises from a combination of systemic fluid restriction, reduced salivary gland output secondary to uremia, and medication side-effects, particularly from phosphate binders and calcimimetics [13,22,30]. Hyposalivation markedly increases the risk of caries, oral candidiasis, and periodontal disease in an already immunocompromised host. The authors noted heterogeneity across studies, so these findings should be interpreted as reported associations rather than uniform clinical profile [12].

Periodontal disease deserves particular attention in CKD patients: a bidirectional relationship between periodontitis and CKD progression is increasingly supported by observational data, with chronic periodontitis contributing to systemic inflammatory burden and potentially accelerating renal functional decline [12]. In our patients, severe generalised periodontitis, heavy calculus deposits, and gingival recession were confluent with the known oral profile of ESRD and long-term dialysis. Uremic fetor, a characteristic ammoniacal malodor arising from salivary conversion of urea to ammonia by oral urease-producing bacteria, was also present, a finding reported in more than half of end-stage hemodialysis patients [12,31].

Renal osteodystrophy (ROD) is the skeletal component of chronic kidney disease–mineral and bone disorder (CKD-MBD), driven by the resulting hyperparathyroidism [32]. The key orofacial radiological features noted on a panoramic radiograph include a generalized reduction in radiodensity affecting both the maxilla and mandible, often accompanied by diffuse swelling of the maxilla and mandible. The trabecular bone pattern, normally sharp and well-defined, is diffusely attenuated, giving the jawbones a smooth, finely granular, “ground-glass” texture rather than the expected coarse trabecular network [29,32]. Cortical margins, including the outline of the inferior mandibular border, the lamina dura surrounding the tooth sockets, and the walls of the maxillary sinus, appear thinned and less radiopaque with the disappearance of the normal radiopaque border at the tooth roots. This is consistent with diffuse bone demineralization secondary to a metabolic bone disturbance [32,33]. Brown tumors that are well-demarcated, unilocular, or multilocular osteolytic lesions are seen at the sites of excessive osteoclastic resorption. These are focal accumulations of fibrovascular tissue and giant cells, which can expand and distort the jaw contour [34]. However, it is notable that on plain radiographs, CTs, and MRIs, brown tumors and amyloid depositions can be difficult to differentiate from a true neoplasm.

The panoramic radiographic findings in our patients, like ground-glass trabecular pattern, loss of lamina dura, and cortical thinning, are well-described hallmarks of secondary hyperparathyroidism affecting the jaw bones, mirroring similar reports in the literature [29,35]. Generalised loss of lamina dura, though not pathognomonic, remains one of the most consistently reported jaw radiographic signs in ROD. The ground-glass appearance reflects the replacement of normal compact trabecular bones by irregular woven bone and fibrous tissue, producing a homogeneous granular opacity [29].

### 3.3. Histopathological Considerations and Differential Diagnosis

The histopathological features of osteitis fibrosa cystica fibrous marrow replacement, widened osteoid seams with irregular mineralization, osteoblastic rimming, and osteoclastic resorption as observed in our case, are consistent with the published series of ROD-related jaw lesions [21,25,27]. Distinguishing ROD from other fibro-osseous jaw lesions requires close clinicopathological correlation of history of CKD and dialysis with serum PTH and mineral-metabolism results, and the radiographic pattern, as histology alone is insufficient [12]. Those features favor micrognathia secondary to renal osteodystrophy. Additional confirmation is provided by correlation with imaging, PTH levels, and histopathology. Brown tumors are usually distinct, well-defined osteolytic lesions that are rich in large cells. A single brown tumor is less likely to cause micrognathia, which exhibits diffuse bilateral growth of the maxilla and mandible, generalized cortical expansion, altered trabeculation, and enlarged facial dimensions [15,16,28]. Fibrous dysplasia may show a similar “Chinese letter” pattern of woven bone trabeculae within a fibrous stroma, though it characteristically lacks the osteoblastic rimming; is typically monostotic and craniofacial in distribution; and is not associated with biochemical evidence of hyperparathyroidism [36]. Paget’s disease of bone may produce similar jaw enlargement and ground-glass radiographic changes, but the characteristic “mosaic” cement lines and markedly elevated serum alkaline phosphatase help differentiate it [36]. Primary hyperparathyroidism was excluded by the clinical context of secondary disease arising from ESRD. These entities must be systematically excluded using clinical and biochemical data before attributing jaw enlargement to ROD.

### 3.4. Reported Cases of Macrognathia in ROD

The literature on ROD-associated macrognathia remains limited. Damm et al., in a multicenter review of nine dialysis patients who exhibited bilateral jaw enlargement with fibro-osseous histopathology, highlighted the radiographic and histopathological overlap with other bone disorders [16]. Hata et al. reported one of the first cases of successful regression of macrognathia following total parathyroidectomy with auto implantation, with immunohistochemical evidence that removal of the PTH drive halted osteoblast and osteoclast activation in the jaw [25]. Benjelloun et al. similarly documented marked facial enlargement secondary to secondary hyperparathyroidism in a dialysis patient [26]. More recently, Demenech et al. (2022) reported a Brazilian female patient with bilateral jaw enlargement and CKD-related secondary hyperparathyroidism [29], and Lerman et al. (2024) documented localized mandibular enlargement in ESRD [8], highlighting the continued clinical relevance of this rare manifestation. The current case adds to this body of literature. To our knowledge, this is among the few cases of ROD-associated macrognathia reported from a Gulf Cooperation Council (GCC) country.

### 3.5. Management Principles

Dental interventions in treating orofacial ROD are in essence an adjunct to the management of the underlying CKD-MBD. Management of ROD is guided by some key principles and must be carried out in coordination with the nephrology team. The primary focus of management is the control of secondary hyperparathyroidism. Managing the risk of hypercalcemia and hyperphosphatemia is a priority; phosphate binders, vitamin D receptor activators (calcitriol, paricalcitol), and calcimimetics remain the first line of therapy to suppress PTH. When these become ineffective, parathyroidectomy is the next possible step. The decrease in bone strength accompanying the loss of PTH, may additionally require treatment using antiosteoporotic drugs [23,37].

The second principle is a significant recent modification in CKD-MBD/ROD management, that focuses on the intravenous use of calcimimetics in combination with biological therapy. It is a shift from oral cinacalcet to intravenous etelcalcetide. In patients who decline to undergo parathyroidectomy or are considered unsuitable for it, a combination therapy with denosumab may be tried. Etelcalcetide is a synthetic calcimimetic peptide that binds to the extracellular domain of the calcium-sensing receptor on parathyroid chief cells. It increases the receptor sensitivity to extracellular calcium, and, therefore, suppresses PTH secretion. Intravenous administration at the end of each dialysis session and lesser gastrointestinal intolerance and CYP2D6 drug interactions, improves patient compliance and adherence in contrast to oral cinacalcet [38]. A study showed a significant reduction in PTH in patients receiving etelcalcetide with some patients having more than 50% drop in PTH levels [39]. Before any elective oral surgery, patients on IV calcimimetic therapy should be monitored for PTH and corrected calcium levels to avoid abrupt biochemical alterations that can affect bone-healing capacity. For patients who decline a clinically indicated parathyroidectomy for refractory secondary hyperparathyroidism, a combination of calcimimetics, vitamin D receptor activators, and denosumab may be used to manage bone and mineral parameters [40].

Experimental studies on rat models with CKD and secondary hyperparathyroidism show that etelcalcetide reduced PTH, improved mineral homeostasis, and prevented excessive bone resorption, thus increasing bone mass [41]. Although data from direct orofacial outcome remain limited, an etelcalcetide-driven decrease in PTH has also been associated with a reduction in ectopic and vascular calcification, plausibly so due to reducing brown tumor formation and jaw cortical thinning [42].

The third principle outlines the surgical approach with subtotal or total parathyroidectomy as the definitive treatment for severe or medically refractory hyperparathyroidism. This surgical intervention halts ongoing bone resorption, and a radiographic regression of brown tumors may be seen over several months post-operatively [37]. As reported by Hata et. al. and Benjelloun et. al., parathyroidectomy is the only known intervention to regress macrognathia linked to ROD patients [25,26].

The fourth principle directs integrated care. According to the KDIGO 2024 Clinical Practice Guidelines, CKD-MBD should be managed as an integrated calcium–phosphate–PTH–vitamin D targeted strategy and not treat hyperparathyroidism in isolation [1]. Jaw lesions should be investigated biochemically and not managed as an isolated local lesion. A personalized plan, guided by serial monitoring of serum phosphate, calcium, PTH levels, and 25-hydroxyvitamin D to control hyperphosphatemia, correct calcitriol deficiency, and suppress secondary hyperparathyroidism, is recommended [23]. Dietary phosphate restriction and use of non-calcium-based phosphate binders such as sevelamer or lanthanum carbonate are favored and restriction of calcium-based binders due to fears of hypercalcemia and vascular calcification [23].

The fifth principle guides the oral—maxilla—facial specific measures. Dental management for ROD patients is mostly supportive and symptomatic. Close collaboration of the nephrology, endocrinology, and oral surgery teams regarding dialysis timing, anticoagulation, and calcium–PTH status before extractions or biopsy of a suspected brown tumor is essential. Histopathological confirmation of expansile jaw lesion must not be assumed as a brown tumor due to radiographic overlap with fibrous dysplasia and true neoplasms. Routine radiographic and clinical monitoring of the jaw lesions is important since regression is dependent on degree of biochemical control. Emphasis is laid on oral prophylaxis, prevention of caries, management of xerostomia, and avoidance of secondary infections, including oral candidiasis. During active or uncontrolled hyperparathyroidism in dialysis patients, unnecessary invasive dental procedures must be avoided due to impaired bone healing and bleeding risk [24,35,38].

Contemporary management of CKD-MBD follows the KDIGO 2017 Clinical Practice Guideline, which advocates a personalized, biochemistry-guided approach to control hyperphosphatasemia, correct calcitriol deficiency, and suppress secondary hyperparathyroidism [7,23]. Serial monitoring of serum phosphate, calcium, intact PTH, and 25-hydroxyvitamin D is recommended; treatment decisions are based on progressive trends rather than single values. Phosphate restriction is achieved through dietary counselling and phosphate binders; the guideline suggests restricting calcium-based binders due to concerns regarding hypercalcemia and vascular calcification in favor of non-calcium-based agents such as sevelamer or lanthanum carbonate [7]. Calcitriol or vitamin D analogues (e.g., paricalcitol) are used to suppress PTH while managing the risk of hypercalcemia and hyperphosphatemia. Calcimimetics (cinacalcet or etelcalcetide in dialysis patients) offer an important adjunct by directly activating the calcium-sensing receptor on parathyroid cells, lowering PTH independent of calcium and phosphate levels [7].

Patients with markedly increased PTH refractory to pharmacological treatment should undergo surgical parathyroidectomy. As shown in the cases by Hata et al. and Benjelloun et al., parathyroidectomy is the only known intervention for the regression of macrognathia linked to ROD patients [14,15]. Dental therapy for these patients is supportive and symptomatic, with an emphasis on oral prophylaxis, caries prevention, xerostomia management, and the avoidance of secondary infections, including oral candidiasis [13,21,24].

## 4. Limitations

The paper describes a single case. Therefore, the findings cannot be generalized to all patients with chronic kidney disease, renal osteodystrophy, or secondary hyperparathyroidism.

Photographs and detailed findings of earlier investigations of the patient prior to the development of the swellings were not available for comparison.

The contribution of individual factors such as duration of dialysis, adherence to various medications, levels of alkaline phosphatase, and parathyroid hormone could not be separately quantified. Therefore, these findings should be interpreted with caution and require confirmation in larger longitudinal studies.

Despite these limitations, this case highlights the importance of early recognition and multidisciplinary management of severe secondary hyperparathyroidism and ROD in hemodialysis patients presenting with craniofacial deformity.

## 5. Conclusions

This case report detailed an uncommon oral presentation of renal osteodystrophy macrognathia in a patient on hemodialysis. Increasing bilateral jaw enlargement with characteristic ground-glass appearance on panoramic radiographic imaging, and histopathological features of osteitis fibrosa cystica in patients with secondary hyperparathyroidism due to long-standing ESRD, is a diagnostic triad of the condition.

Some key points are emphasized by this case. Firstly, as dental clinics often are the first point of care for unexplained jaw enlargement, dentists should have a high index of suspicion for an underlying systemic metabolic derangement in such patients, especially in known cases of CKD or those on long-term dialysis. Secondly, thorough oral examination, including evaluation for mucosal pallor, halitosis, xerostomia, condition of the periodontal tissue, and bone changes on radiography, offer important pointers in CKD patients. Thirdly, interdisciplinary cooperation between oral health specialists, nephrologists, and endocrinologists is essential for efficient management of such a specific subgroup of patients. Fourthly, maintenance of dental health is necessary to reduce further morbidity by routine professional debridement, proactive and individualized oral hygiene training, caries prevention, and antifungal prophylaxis in an already immunocompromised and complex systemic disease state.

Despite biochemical improvement after medical management of CKD-MBD, jaw enlargement caused by ROD may continue to persist. In such resistant cases of secondary hyperparathyroidism, the most effective way to stop or reverse bone growth is parathyroidectomy. Though uncommon, timely reporting of such cases, especially outcomes of long-term follow-up, is essential in reiterating the early identification and management of oral lesions due to metabolic derangements in CKD.

## Figures and Tables

**Table 1:** Clinical and radiological findings of differential diagnoses.

Condition	Findings for	Findings Against
Diffuse ROD-associated macrognathia	Long-term HD; severe secondary HPT; bilateral, symmetrical enlargement of both jaws; generalised ground-glass pattern; loss of lamina dura and cortices	-
Localized brown tumor	Secondary HPT	No discrete, well-defined unilocular or multilocular radiolucency; enlargement diffuse, not focal
Fibrous dysplasia	Ground-glass pattern	Onset usually in childhood or adolescence; usually unilateral and regional, not generalised; no biochemical HPT
Central giant cell granuloma	Giant cells may occur in ROD	Focal, well-defined radiolucency; not bilateral or diffuse
Ossifying fibroma	Painless jaw expansion	Well-demarcated lesion with a radiolucent rim; focal
Paget’s disease	Jaw enlargement; raised ALP	Age 45; mandible-predominant site; no “cotton wool” appearance; no skull involvement; HPT explains ALP rise
Malignant bone neoplasm	Progressive swelling	No pain, paresthesia, ulceration, rapid growth, cortical destruction or periosteal reaction

**Table 2:** Matching patient characteristics to features of Sagliker syndrome.

Sagliker Syndrome Feature	This Patient
Maxillary and mandibular overgrowth (“uglifying” facial change)	Present
Soft-tissue masses in the mouth	Present (palatal swelling)
Dental abnormalities (wide spacing, malposition)	Present
Fingertip changes (clubbing, acro- osteolysis)	Absent
Short stature	Absent- height 162 cm
Knee and skull changes	Absent
Hearing abnormalities	Absent
Neurological or psychological problems	Absent

**Table 3:** Laboratory investigations at presentation.

Parameter	Unit	Reference Range	Presentation
Intact PTH	pg/mL	15–65	1200
Corrected calcium	mmol/L	2.15–2.55	2.05
Phosphate	mmol/L	0.81–1.45	2.42
Alkaline phosphatase	U/L	40–129	412
25-hydroxyvitamin D	ng/mL	30–100	12
Hemoglobin	g/dL	12.0–15.5	9.1
Bicarbonate	mmol/L	22–29	17
Albumin	g/L	35–52	31
Ferritin (ng/mL)/TSAT (%)		-	620/18

**Table 4:** Laboratory investigations at three time points.

Parameter	Unit	Reference Range	Time Points of Testing		
			Presentation	3 Months	Latest (12 Months)
Intact PTH	pg/mL	15–65	1,200	480	210
Corrected calcium	mmol/L	2.15–2.55	2.05	2.28	2.35
Phosphate	mmol/L	0.81–1.45	2.42	1.68	1.42
Alkaline phosphatase	U/L	40–129	412	268	154
25-hydroxyvitamin D	ng/mL	30–100	12	38	46
Hemoglobin	g/dL	12.0–15.5	9.1	10.8	11.6
Bicarbonate	mmol/L	22–29	17	22	24
Albumin	g/L	35–52	31	36	39
Ferritin (ng/mL)/TSAT (%)		-	620/18	540/24	480/28

## Data Availability

Data is contained within the article.

## Abbreviations

The following abbreviations are used in this manuscript:

CKD	Chronic Kidney Disease
ESRD	End- Stage Renal Disease
ROD	Renal OsteoDystrophy
CKD-MBD	Chronic Kidney Disease—Metabolic Bone Disease
PTH	ParaThyroid Hormone
CBCT	Cone Beam Computed Tomography
KDIGO	Kidney Disease—Improving Global Outcomes
CYP	Cytochrome P
FGF	Fibroblast Growth Factor
FGFR	Fibroblast Growth Factor Receptor
RANK	Receptor Activator of Nuclear factor Kappa-B
RANKL	Receptor Activator of Nuclear factor Kappa-B Ligand
